# B7‐H3 protein expression in acute myeloid leukemia

**DOI:** 10.1002/cam4.522

**Published:** 2015-09-17

**Authors:** Thomas Guery, Christophe Roumier, Celine Berthon, Aline Renneville, Claude Preudhomme, Bruno Quesnel

**Affiliations:** ^1^ Laboratoire d'HématologieCHRULilleFrance; ^2^ INSERMUMR 1172LilleFrance; ^3^ Service des Maladies du SangCHRULilleFrance; ^4^ Université de LilleLilleFrance

**Keywords:** AML, B7‐H3, CD276, CEBPA, NPM1, prognosis

## Abstract

Costimulatory molecules are essential regulators of the immunological synapse and enable the fine‐tuning of the immune response. These mechanisms are subverted by cancer cells to evade immunosurveillance. The B7 family of costimulatory molecules comprises several ligands that may contribute to immunoescape. B7‐H3 [B7‐homolog 3 or CD276] remains poorly investigated in hematological malignancies. To determine the role B7‐H3, we analyzed the expression of this molecule in blast cells from a cohort of 111 acute myeloid leukemia (AML) patients. B7‐H3 was expressed in blast cells with a mean fluorescence intensity ratio >3 in 30 (27%) of the 111 patients. B7‐H3 expression was higher in the M3 and M5 FAB subtypes and in cases with mutated *NPM1* and wild type *CEBPA*. There were no significant differences found for the FLT3‐ITD or cytogenetic risk groups. The complete remission (CR) rate between the 17 B7‐H3‐positive and 58 negative patients who were treated intensively was not different. The event free survival was longer in B7‐H3‐positive patients (*P* = 0.014), and there was a trend toward better overall survival. However, this difference was not statistically significant (*P* = 0.053). In conclusion, B7‐H3 is one of the most strongly expressed B7‐family molecules in AML and merits further investigation.

## To the Editor

Costimulatory molecules are essential regulators of the immunological synapse. The fine tuning of the immune response requires both positive and negative signals to ensure efficient responses against pathogens and the appropriate termination of immune system activation to avoid tissue damage. These mechanisms are subverted by cancer cells to evade immunosurveillance. The B7 family of costimulatory molecules comprises several ligands that may contribute to immunoescape, including PD‐L1 (B7‐H1) and B7‐H4, and inhibitory receptors such as PD‐1 and CTLA4 1. These molecules have been extensively studied in cancers, including acute myeloid leukemia (AML), and efficient therapeutic monoclonal antibodies have been developed 2.

The role in cancer of B7‐H3 [B7‐homolog 3 or CD276], another member of the B7 family, is less clear 3. B7‐H3 is expressed in many tissues and on antigen‐presenting cells. In peripheral blood cells, the level of basal expression appears to be low but increases in monocytes after exposure to lipopolysaccharide (LPS) and stimulation by interferon‐*γ*. The functions and counter receptor of B7‐H3 remain unclear. B7‐H3 has been reported to be an enhancer of the T‐cell response, but other reports have described a suppressive role. The expression of the B7‐H3 protein by cells in the tumor microenvironment and by tumor cells modifies the antitumor immune reaction, tumor growth, metastatic ability and drug resistance. B7‐H3 may suppress NK cells through an unidentified receptor. B7‐H3 is widely expressed in solid cancers and seems to be a prognostic marker in several tumor types 3.

The role of B7‐H3 and its expression in AML have not yet been extensively investigated. To address the question of a possible role of B7‐H3, we analyzed the expression of this molecule in blast cells from a cohort of 111 AML patients. The median age of the patients was 49 (0–88) years, with 18 children and 93 adults. Forty‐five patients had leukocytosis, >20 G/L; 96 patients had primary AML, and 15 had AML secondary to myelodysplastic or myeloproliferative syndromes.

Bone marrow mononuclear cells were isolated via Ficoll‐Hypaque centrifugation after the donors had given informed consent in accordance with the Declaration of Helsinki. This study was approved by the Institutional Review Board of Tumorotheque/CHU Lille. The expression of the B7‐H3 molecule was evaluated by flow cytometry with an anti‐B7‐H3‐PE monoclonal antibody (mAb) (R&D Systems, Minneapolis, MN) in blast cells gated with an anti‐CD45‐PC5 mAb (Beckman Coulter, Miami, FL). The B7‐H3 mean fluorescence intensity (MFI) of lymphocytes, monocytes, total blast cells and CD34+, CD34+/CD38− and CD34+/CD38−/CD123+ subfractions was evaluated. Blasts were considered positive when the B7‐H3 MFI blasts/lymphocytes ratio was greater than 3. Fifteen nonleukemic bone marrow samples were used as controls.

**Figure 1:**
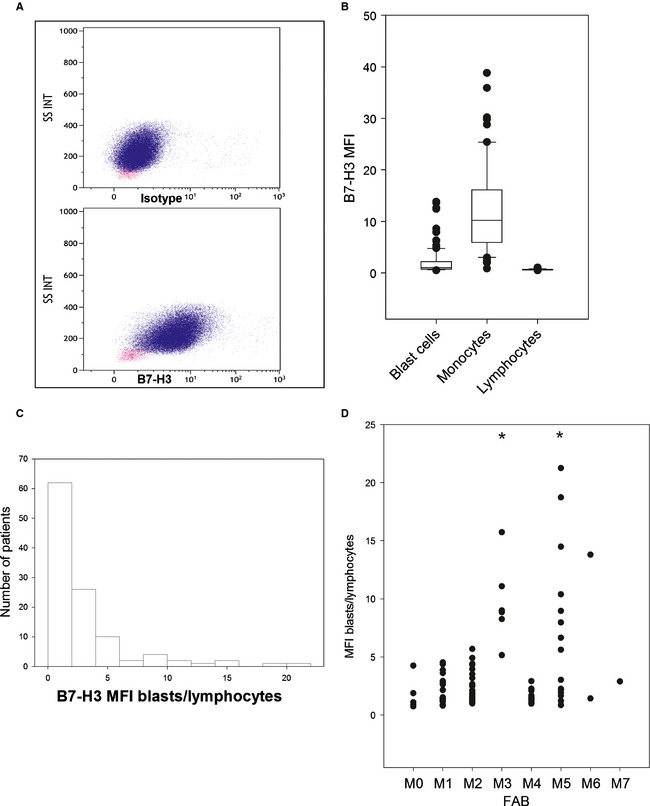
B7‐H3 expression in blast cells from patients with AML. (A) Flow cytometry analysis of B7‐H3 expression in a representative sample of blast cells. (B) B7‐H3 MFI in blast cells, monocytes, and lymphocytes in the cohort of 111 patients with AML. (C) Distribution of B7‐H3 blast/lymphocytes MFI in the same cohort as (B). (D) B7‐H3 MFI blasts/lymphocytes according to FAB type. *P < 0.001.

**Figure 2:**
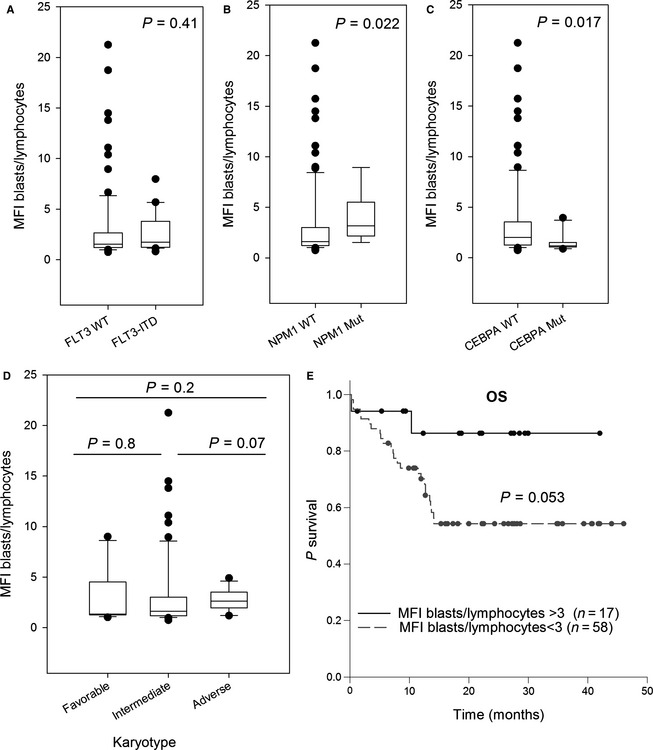
B7‐H3 expression according to patient characteristics. B7‐H3 MFI blasts/lymphocytes according to *FLT3*‐ITD (A), *NPM1*. (B), and *CEBPA* (C) mutational status. (D) Same as (A) but for karyotype. (E) Overall survival of the 75 patients treated intensively according to a B7‐H3 MFI blasts/lymphocytes threshold at 3.

The expression of B7‐H3 in lymphocytes appeared to be weak and stable (MFI: 0.678, SD: 0.099) across samples (Fig. 1). We then decided to measure and compare B7‐H3 expression through the MFI blasts/lymphocytes ratio. Monocytes exhibited heterogeneous expression at higher levels. B7‐H3 was expressed in blast cells with an MFI ratio >3 in 30 (27%) of the 111 patients. B7‐H3 expression did not follow a normal distribution pattern (Fig. 1C). No significant differences between the CD34+/CD38−, CD34+/CD38+, and CD34+/CD38−/CD123+ subfractions were observed (data not shown). B7‐H3 expression was higher in the M3 and M5 FAB subtypes (Fig. 1D). No correlation was observed between B7‐H3 and sex, age, leukocytosis, bone marrow blast percentage, or cytogenetic risk group (Fig. 2). Cases in which *NPM1* was mutated and *CEBPΑ* was wild‐type showed higher B7‐H3 expression (Fig. 2B and C). No significant differences were seen for FLT3‐ITD (Fig. 2A). Because B7‐H3 has been reported to be a prognostic marker in certain solid tumors and in a recent leukemia cohort 4, we compared the complete remission (CR) rates and overall survival (OS) between the 17 B7‐H3‐positive and 58 negative patients who were treated intensively. No difference in the CR rates (81 vs. 89% in B7‐H3‐positive vs. negative AML patients, *P* = 0.16) was found. The event‐free survival was significantly better in B7‐H3‐positive patients (*P* = 0.014) (Fig. S1). However, there was only a trend toward better OS in the B7‐H3‐positive patients, and this difference was not statistically significant (*P* = 0.053) (Fig. 2E).

The question of the role of the B7 family of molecules in AML remains controversial. This controversy is related to the variable expression levels reported across studies, which is likely a consequence of the inducible expression characteristics of these proteins. We previously reported that PD‐L1 expression was observed in AML blast cells after exposure to Toll‐like receptor (TLR) ligands or IFN*γ*. However, the basal levels were high in a minority of patients 2. Similar findings were observed for B7.1 (CD80) and B7.2 (CD86), which can be induced by oxidative stress 5. Currently, only B7.2 is associated with a worse prognosis, but this finding remains controversial 6. B7‐DC and B7‐H4 expression were not detected. In this study, B7‐H3 expression in AML blast cells appeared to be high under basal conditions in a large proportion of the patients. This result suggests that B7‐H3 protein is one of the few strongly expressed B7‐family molecules in myeloid leukemia cells. This high expression level was frequently observed in *NPM1*‐mutated AML patients and in patients with M5 subtypes. These findings suggest B7‐H3 expression is a characteristic of the monocytic lineage, and it is observed in the monocytes of healthy controls. A recent publication reported a negative prognostic impact of B7‐H3 expression in a cohort of acute leukemia patients and higher expression levels in the patients with adverse karyotypes 4. However, the studied cohort comprised both lymphoid and myeloid leukemia patients, and the proportion of patients receiving intensive chemotherapy was not indicated. In this study, there was no significant difference in OS according to B7‐H3 expression and there was no correlation with the cytogenetic risk group. Moreover, several AML subsets overexpressing B7‐H3 had good features (*NPM1* mutations, APL). These results suggest a large patient cohort would be required to establish the prognostic significance of B7‐H3 expression regarding the OS.

In conclusion, B7‐H3 is one of the most strongly expressed B7‐family molecules in AML, and further exploration is merited to clarify the role of B7‐H3 in myeloid malignancies.

## Conflict of Interest

None declared.

## Supporting information


**Figure S1.** Event‐free survival of the 75 patients treated intensively according to a B7‐H3 MFI blasts/lymphocytes threshold at 3.
