# Supplementary material for: B7‐H3 protein expression in acute myeloid leukemia
Source: Cancer Med. 2015 Sep 17;4(12):1879–83. doi: 10.1002/cam4.522 (PMC5123710; doi:10.1002/cam4.522)
Supplement: Supplementary file 1 — Figure S1. Event‐free survival of the 75 patients treated intensively according to a B7‐H3 MFI blasts/lymphocytes threshold at 3. [file CAM4-4-1879-s001.pdf]

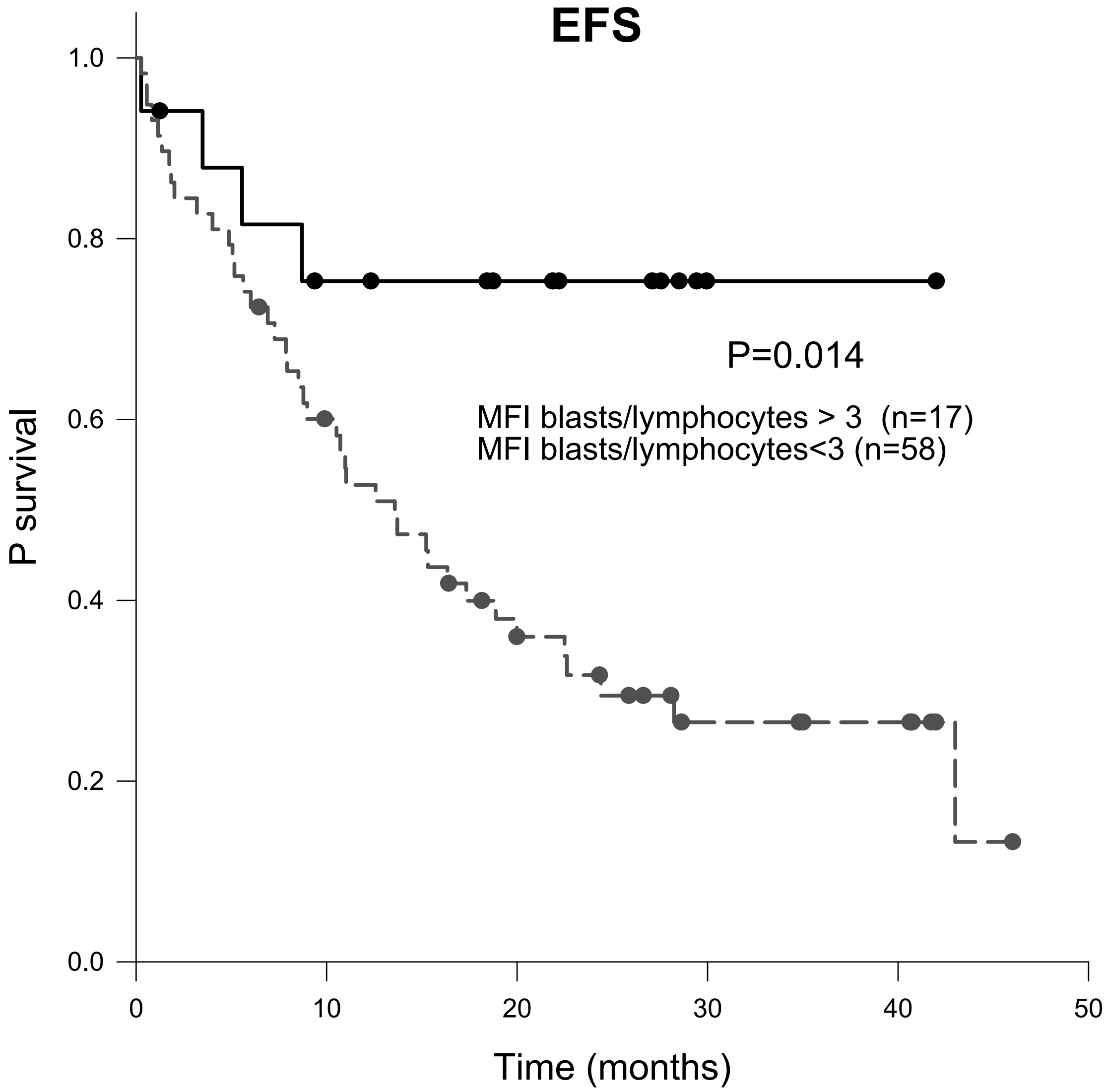

Supplementary Figure S1: Event free survival of the 75 patients treated intensively according to a B7-H3 MFI blasts/lymphocytes threshold at 3
